# A randomised trial of a contraceptive vaginal ring in women at risk of HIV infection in Rwanda: Safety of intermittent and continuous use

**DOI:** 10.1371/journal.pone.0197572

**Published:** 2018-06-01

**Authors:** Evelyne Kestelyn, Stephen Agaba, Jennifer Ilo Van Nuil, Mireille Uwineza, Marie Michelle Umulisa, Lambert Mwambarangwe, Jean Claude Ndagijimana, Irith De Baetselier, Jozefien Buyze, Thérèse Delvaux, Tania Crucitti, Vicky Jespers, Janneke H. H. M. van de Wijgert, for the Ring Plus Study Group

**Affiliations:** 1 Rinda Ubuzima, Kigali Health Institute, Kigali, Rwanda; 2 Department of Clinical Infection, Microbiology and Immunology, Institute of Infection and Global Health, University of Liverpool, Liverpool, United Kingdom; 3 Institute of Tropical Medicine, Antwerp, Belgium; IAVI, UNITED STATES

## Abstract

**Background:**

Contraceptive vaginal rings could play a role in expanding the contraceptive method mix and in preparing communities for the introduction of HIV prevention and multipurpose rings.

**Methods:**

We conducted an open label single-centre randomised clinical trial of intermittent versus continuous use of NuvaRing® in Kigali, Rwanda, in 2013–2014. We randomised 120 HIV-negative women 1:1 to intermittent use (three rings with a ring-free week in between rings) or continuous use (four rings without ring-free weeks). Women underwent an interview, counselling, and a speculum examination, and were tested for pregnancy, bacterial vaginosis (BV) by Nugent scoring, yeasts and trichomonads on wet mount, and sexually transmitted infections.

**Findings:**

Only one woman withdrew early. Deliberate ring removals were rare, but spontaneous ring expulsions occurred during 14% of ring use periods. There were no incident pregnancies, serious adverse events, serious social harms, or early discontinuations for safety reasons. Systemic side effects were uncommon, and local side effects were not significantly differently distributed between groups except for lower abdominal pain (*P* = 0.013). The incidence of vaginal yeasts during ring use was high: 22% of intermittent users and 27% of continuous users had incident vaginal yeasts at one or multiple ring removal visits (*P* = 0.666), and symptomatic vaginal yeast cases were more common in the continuous than intermittent users (*P* = 0.031). In contrast, mean Nugent scores improved over time in both groups.

**Conclusions:**

Intermittent and continuous NuvaRing® use were safe in Rwandan women and improved Nugent scores over time. However, attention should be paid to ring expulsions and to a potential increased risk of vaginal candidiasis.

## Introduction

Vaginal rings are polymeric drug delivery devices designed to provide controlled release of drugs for vaginal administration over extended periods of time. Compared to systemic dosing, the sustained local drug release over a period of several weeks maximises efficacy at lower doses as well as adherence [1]. For these and other reasons, vaginal rings have become popular for contraception and oestrogen replacement therapy in Europe, the United States (US), and Latin America [1]. The contraceptive vaginal ring NuvaRing® (containing an oestrogen and a progestin) is available most widely; one ring is licensed to remain in the vagina for three weeks, followed by one ring-free week to allow for withdrawal bleeding [2–4]. Progering® (containing progesterone only) is currently only marketed in South America for breastfeeding women; one ring is licensed to remain in the vagina for up to three months [5]. These contraceptive vaginal rings are currently not routinely available in public clinics in any sub-Saharan African country despite the large burden of unplanned pregnancies in many of those countries [6].

More recently, vaginal rings releasing antiretroviral drugs have been developed for HIV prevention, and two Phase 3 efficacy trials of the dapivirine vaginal ring (International Partnership for Microbicides, Silver Spring, MD US) showed a protective effect in sub-Saharan African women at risk of HIV infection [7, 8]. The expectation is that vaginal rings for HIV prevention, as well as vaginal rings for HIV and pregnancy prevention (so-called multipurpose rings), will continue to be developed, and will eventually be rolled out in HIV-endemic areas including sub-Saharan Africa [9]. Such rings will have to be used continuously, which is different from NuvaRing®’s currently labelled use.

The Rwandan government has prioritised family planning and HIV prevention in the last decade. The total fertility rate in Rwanda declined from 6.1 children per woman in 2005 to 4.5 in 2016, and the aim is to reduce this further to 3.0 children per woman [10]. Several reliable family planning methods are already available in public clinics. However, the most popular method is the Depo Provera® injection, which has been associated with an increased risk of HIV acquisition in several observational studies [11]. The Rwandan government is committed to strengthening its family planning program further, and also showed its commitment to expanding HIV prevention options for women by hosting several clinical trials of vaginal gels for HIV prevention between 2005 and 2012 [12].

We conducted a randomised clinical trial of intermittent versus continuous use of NuvaRing® in HIV-negative women at risk of HIV in Kigali, Rwanda [13]. The main objective of this trial was to confirm safety in this setting, in preparation for the potential roll out of vaginal rings for contraception and/or HIV prevention in Rwanda in the future. We also evaluated the effects of ring use on the vaginal microbiota and biofilm formation, and conducted in-depth mixed methods research on ring use acceptability and adherence, but this paper focusses on the safety results.

## Methods

### Study design, endpoints, and ethical review

The study design was an open label single-centre randomised clinical trial of intermittent versus continuous use of NuvaRing® (S1 Fig, S2 Fig). Each ring was inserted for three weeks. A total of 120 HIV-negative women were randomised 1:1 to intermittent use (three rings with a ring-free week in between rings) or continuous use (four consecutive rings without ring-free weeks). Adherence was assessed by quantifying ring removals and expulsions. Safety endpoints included (serious) adverse events (AEs), (serious) social harms, early discontinuation of ring use for safety reasons, self-reported urogenital symptoms, clinician-observed signs during pelvic and bimanual examinations, and laboratory-confirmed reproductive tract infections (with an emphasis on bacterial vaginosis (BV) and vaginal candidiasis). Acceptability (including cycle control) findings are briefly summarised to provide a context for the safety results but will be reported in more detail elsewhere.

The trial was sponsored by the Institute of Tropical Medicine (ITM) in Antwerp, Belgium, registered in clinicaltrials.gov (NCT01796613), and conducted in accordance with the Good Clinical (and Laboratory) Practices guidelines and the Declaration of Helsinki [13]. It was approved by the National Health Research Committee of the Rwanda Ministry of Health, the Rwanda National Ethics Committee, and the ethics committees of ITM, the University of Antwerp, and the University of Liverpool. All participants provided written informed consent. We obtained additional written consent from independent witnesses in the case of illiterate participants, and from parents or guardians in the case of participants aged 18–21, which was in agreement with the Rwandan regulations at the time of the study.

### NuvaRing®

NuvaRing® is manufactured by Organon (a subsidiary of Merck & Co), Oss, Netherlands. It is a thin (cross-section 4 mm), soft, flexible and transparent ring containing 11.7 mg etonogestrel and 2.7 mg ethinylestradiol [2–4]. It releases an average amount of 0.120 mg etonogestrel and 0.015 mg ethinylestradiol per 24 hours and is licensed for three weeks of use followed by a ring-free week. Product labelling states that if the ring is outside the vagina for longer than three hours it may be reinserted but a backup contraceptive method should be used for seven days. During the trial, rings were kept at 2–8°C but brought to room temperature prior to vaginal insertion.

### Study participants

To be eligible, women had to be 18–35 years old, be generally in good physical and mental health, and test negative for HIV and pregnancy at screening. They should currently not be using a modern contraceptive method (with the exception of barrier methods) but be interested in initiating NuvaRing® use. Women were excluded if they had used hormonal contraception in the three months prior to screening; were currently smoking, breastfeeding, or using antimicrobial medication; or had a (history of a) condition contraindicating NuvaRing® use (hysterectomy, recent genital tract surgery, significant urogenital or uterine prolapse, undiagnosed vaginal bleeding, incontinence, chronic and/or recurrent vulvovaginal candidiasis, urethral obstruction, cardiovascular disease, venous thrombosis, migraine with focal neurological symptoms, diabetes mellitus with vascular involvement, pancreatitis, severe hepatic disease, or known/suspected hypersensitivity to any of the NuvaRing® excipients).

### Recruitment and screening procedures

Study staff and community mobilisers contacted women who had participated in previous studies at Rinda Ubuzima (RU) and organised recruitment meetings in Kigali with the prior written approval of local authorities. Potentially eligible women were given an appointment for screening at the RU research clinic and laboratory in Kigali, Rwanda. At the first screening visit, after written informed consent for screening had been obtained, contact- and menses information were collected and HIV/STI and family planning counselling were done. If still interested and potentially eligible, a second screening visit (referred to as the baseline visit) was scheduled within six weeks after the first screening visit. At that visit, women underwent a face-to-face interview, counselling, rapid HIV and urine pregnancy testing, a physical and pelvic/bimanual examination, and sample collection for STI and vaginal infection testing. If still eligible at the end of that visit, an enrolment visit was scheduled on the first or second day of the participant’s next menses (no visit window allowed). After written informed consent for enrolment had been obtained, another urine pregnancy test and physical examination were conducted, all baseline test results and other medical eligibility criteria were reviewed, and final eligibility was determined. All women received treatment for curable STIs, symptomatic vaginal infections, and condoms free of charge at the baseline visit and (if enrolled) throughout the study. Women requiring other care were referred to public services in their own communities as applicable; Rwanda has good access to antenatal, HIV, and family planning services.

### Randomisation and follow-up procedures

At the enrolment visit (week 1), the woman inserted her first ring while being observed by a study nurse, who subsequently randomised her to intermittent or continuous use by opening the next sealed envelope. The random allocation sequence and envelopes were created at ITM. All subsequent follow-up visits were scheduled to coincide with ring insertions and removals (which were directly observed at the study clinic) and most data collection took place at the ring removal visits [13]. In the intermittent group, visits took place at week 4 (first ring out), week 5 (second ring in), week 8 (second ring out), week 9 (third ring in), and week 12 (third ring out; final ring removal visit). In the continuous group, visits took place at week 4 (first ring out, second ring in), week 7 (second ring out, third ring in), week 10 (third ring out, fourth ring in), and week 13 (fourth ring out; final ring removal visit). Study procedures at all ring removal visits consisted of a face-to-face interview (including acceptability questions and questions about unscheduled ring removals/expulsions and reinsertions), ring adherence and AE/social harm reporting and counselling, urine pregnancy testing, physical and pelvic/bimanual examination, and sample collection for vaginal infection testing. At the final ring removal visit, optional HIV testing and cervical cancer screening by visual inspection of the cervix with acetic acid (VIA) were offered to all participants. Stand-alone ring insertion visits in the intermittent use group were kept brief and consisted of ring insertion and AE/social harm reporting and counselling only.

### Laboratory testing

Blood was tested for HIV by Determine Alere HIV1/2 test (Abbott Diagnostic Division, Hoofddorp, Netherlands), followed by Uni-Gold HIV (Trinity Biotech, Berkeley Heights, NJ US) when the first test was reactive, and Vironostika HIV Uni-Form II Ag/Ab enzyme-linked immunosorbent assay (ELISA) (bioMérieux, Marcy l'Etoile, France) when a tiebreaker was needed. Blood was also tested for herpes simplex virus type 2 (HSV-2) by Kalon HSV-2 gG2 ELISA (Kalon Biological, Guildford, UK) and syphilis by rapid plasma reagin (RPR) test followed by *Treponema pallidum* hemagglutination assay (TPHA) if RPR-positive (both Spinreact Reactivos, Girona, Spain). Urine was tested for pregnancy using the QuickVue (Quidel Corporation, San Diego, CA US) or One Step human chorionic gonadotropin tests (Wondfo, Willibrook, IL US). Vaginal swabs were used to prepare a wet mount and a Gram stain slide for Nugent scoring [14]. KOH was added to the wet mounts to visualise yeasts. The vaginal pH was measured during pelvic examinations by pressing a pH paper strip against the vaginal wall (pH range 3.6–6.1 with 0.3 or 0.4 increments; Dosatest, VWR International, Lutterworth, UK). Real-time PCR testing for *Chlamydia trachomatis* and *Neisseria gonorrhoeae* was conducted at the National Reference Laboratory in Kigali using the Presto CT/NG kit (Goffin Molecular Technologies, Houten, Netherlands).

### Statistical analysis

Data were entered into OpenClinica (OpenClinica LLC, Waltham, MA USA) and Microsoft Access (Microsoft Corporation, Redmond, WA US) databases and analysed using Stata versions 12.0 and 13.0 (Stata Corporation, College Station, TX US). The sample size was based on the vaginal microbiota endpoints (not reported in this paper): we required 95% power to detect a pre-post ring use change of 0.5 log_10_ in *Lactobacillus* genus count, assuming a standard deviation of one log_10_, within each randomisation group. All women who inserted a ring at the enrolment visit were included in the analysis in the group that they were randomised to regardless of adherence. Baseline characteristics are presented as medians and interquartile ranges (IQRs) for continuous data, and counts and percentages for categorical data, for each randomisation group.

AEs fell into two overall categories: those that were structurally assessed at study visits (laboratory-confirmed infections, participant-reported urogenital symptoms, and clinician-observed urogenital signs) and those that were spontaneously reported by asking the participant, prior to the structural assessments, if they experienced an AE or social harm since their last visit. AE type, severity, and relatedness to ring use were determined by study physicians in Rwanda. All AEs were subsequently reviewed and coded by a physician at ITM (VJ) prior to analysis, using the Medical Dictionary for Regulatory Activities (MedDRA) preferred terms [15]. However, urogenital symptoms and signs were coded in more detail than is possible with MedDRA preferred terms to reflect the focus of the trial, and those related to normal menstrual or withdrawal bleeding were not included. In addition, the same ITM physician manually compared each urogenital symptom and sign against laboratory-confirmed infections and prescribed medications to determine if they were or were not associated with a laboratory-confirmed infection. Those that were deemed caused by a laboratory-confirmed infection were reported as a symptomatic infection and not as a separate AE. Symptomatic and asymptomatic laboratory-confirmed infections, structurally assessed urogenital signs and symptoms (not including those associated with a symptomatic infection), and spontaneously reported AEs (not including the former two categories) are presented separately.

The number of women with an incident pregnancy or AE over the full ring use period in each randomisation group were compared using Fisher’s exact tests. Incident cases of pregnancy, vaginal yeasts, trichomoniasis, and HIV were defined as a positive diagnostic test result between the baseline and the last ring removal visits in women who had a negative test result at baseline. In the case of BV, an incident case was defined as having a Nugent score of 0–3 at baseline and a Nugent score of 7–10 at any visit after insertion of the first ring. Because the BV prevalence at baseline was high, asymptomatic BV was not treated, and the nature of BV is transient, we also calculated the mean Nugent scores (with 95% confidence intervals) at each ring removal visit for each randomisation group and plotted these in a line graph. The number of women reporting AEs, as well as the number of reported AEs, were calculated for the entire ring use period, but also as a projected rate per 100 rings used. The reason for this was that continuous users compared to intermittent users used a total of four instead of three rings and AEs were ascertained at the four instead of three corresponding ring removal visits. The rates in each group were compared by Poisson regression with the log number of rings as an offset.

## Results

### Participant flow

**Fig 1:**
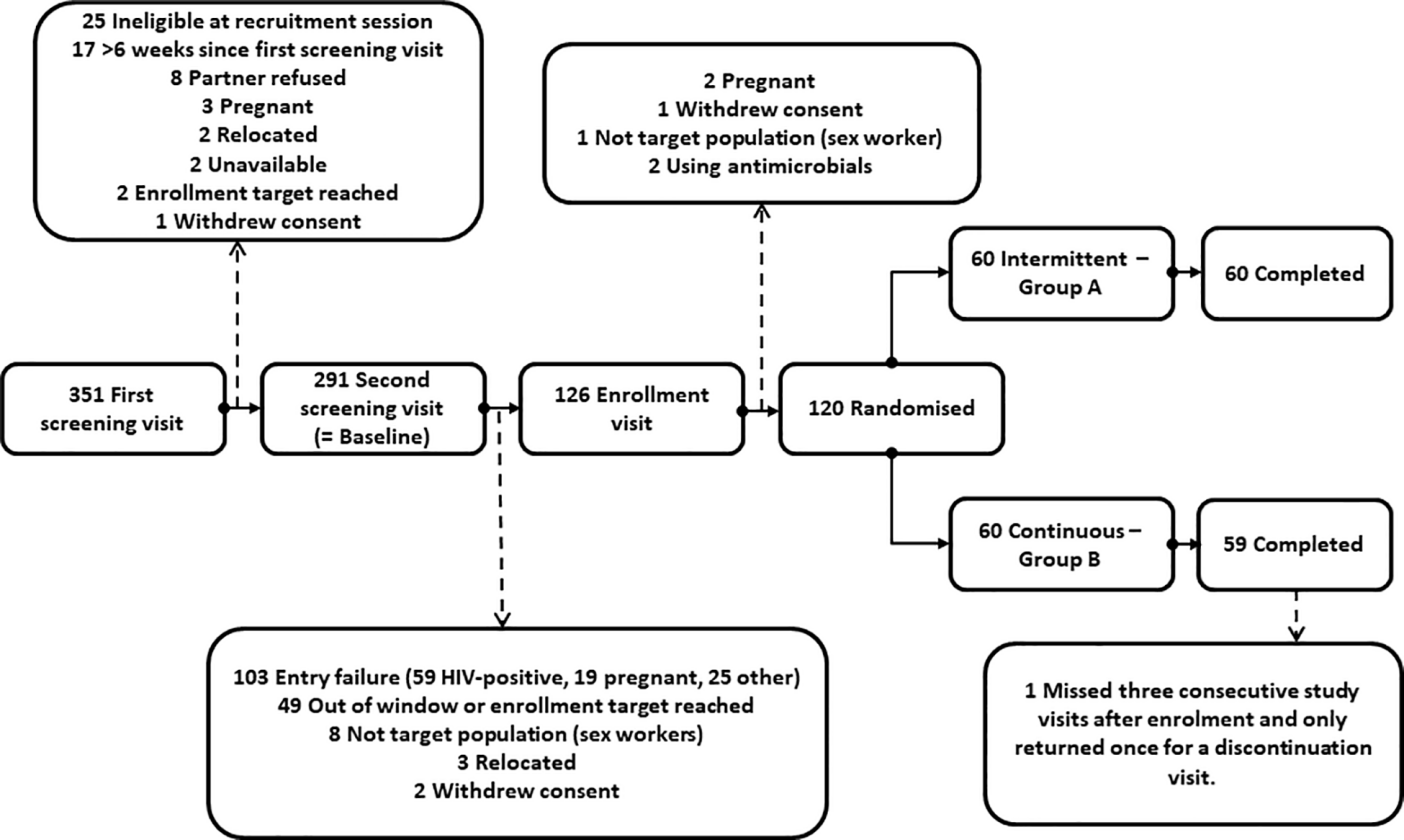
Participant flow.

Between June 2013 and January 2014, we screened 351 women and randomised 120 eligible women (Fig 1). The most common reasons for ineligibility were being HIV-positive (59 women) or pregnant (24 women). Sixty women were randomised to each group, and all but one woman (in the continuous use group) completed the study. One participant was considered discontinued after her first ring because she missed three consecutive study visits and only returned once, several weeks later, for a discontinuation visit. No other study visits were missed, making the total number of ring removal visits 416. Data collection was completed in March 2014.

### Baseline characteristics

The median age of the total screened population and intermittent users was 28 years and of the continuous users 29 years (Table 1). Most women in the screened and enrolled populations had (some) primary school education (65–68%) and earned an income (57–62%). Almost all women in both populations were married or had a regular partner, and had had at least one pregnancy. Even though none of the women were currently using a modern method of contraception, consistent condom use was rare with only 38–39% of the women reporting to have used a condom during their last sex act. The majority of women had used hormonal contraception in the past, with injectables being the most popular method (46% of the screened population, 53% of intermittent users and 45% of continuous users), followed by combined oral contraceptive pills (22%, 18% and 30%, respectively). We recruited women at above-average risk of HIV/STIs: the HIV prevalence was 21%, which is about three times that of the general population of women in Kigali during the study period [16], and other STIs were also common (Table 1). None of the enrolled women had HIV at baseline by design, but the other STI prevalences were similar to those in the screened population.

**Table 1:** Baseline characteristics of the screened population and enrolled population by randomisation group.

Baseline characteristics n (%)	Screened		Intermittent use (N = 60)	Continuous use (N = 60)
	N^1^	n (%)		
Age in years (median, IQR)	347	28 (20–35)	28 (26–31)	29 (26–32)
Education:				
No schooling	347	43 (12)	9 (15)	6 (10)
Primary school^2^		230 (66)	39 (65)	41 (68)
Secondary school^2^		68 (20)	10 (16)	11 (18)
More than secondary school		6 (2)	2 (3)	2 (3)
Earns own income^3^	289	170 (59)	37 (62)	34 (57)
Partnership:				
Married	347	213 (61)	37 (62)	36 (60)
Not married but living together		88 (25)	16 (27)	16 (27)
Not married, regular partner but not living together		42 (12)	7 (12)	8 (13)
No regular partner		4 (1)	0	0
Had additional sex partners in last 3 months	275	16 (6)	2 (3)	2 (3)
Lifetime sex partners:				
1–3	289	233 (81)	51 (85)	53 (88)
4 or more (range: 4–300)		56 (19)	9 (15)	7 (12)
Condom use in last three weeks				
Always	288	42 (15)	12 (20)	9 (15)
Sometimes		155 (54)	24 (41)	35 (58)
Never		91 (32)	23 (38)	16 (27)
Used condom during last sex act	289	113 (39)	23 (38)	23 (38)
Pregnancies:				
0	289	8 (3)	2 (3)	3 (5)
1		57 (20)	12 (20)	10 (17)
2		89 (31)	22 (37)	17 (28)
3 or more (range: 3–7)		135 (47)	24 (40)	30 (50)
Any vaginal deliveries	289	244 (84)	55 (92)	51 (85)
Any Caesarean sections	289	44 (15)	6 (10)	11 (18)
Any past contraceptive use^4^	289	180 (62)	41 (68)	38 (63)
Injectables	289	134 (46)	32 (53)	27 (45)
Contraceptive pills	289	64 (22)	11 (18)	18 (30)
Copper intra uterine device	289	2 (1)	1 (2)	0
Pregnancy test positive	285	19 (7)	0	0
BV^5^:Nugent score 0–3	185	82 (44)	24 (40)	24 (41)
Nugent score 4–6		22 (12)	7 (12)	7 (12)
Nugent score 7–10		81 (44)	29 (48)	28 (47)
Yeasts on wet mount	186	11 (6)	2 (3)	4 (7)
Trichomonads on wet mount	186	13 (7)	5 (8)	4 (7)
HIV by algorithm	285	59 (21)	0	0
HSV-2 serology	285	149 (52)	21 (35)	26 (43)
Syphilis serology	285	25 (9)	3 (5)	3 (5)
Chlamydia PCR	186	15 (8)	4 (7)	6 (10)
Gonorrhoea PCR	186	15 (8)	2 (3)	5 (8)

BV = bacterial vaginosis; IQR = interquartile range; HIV = human immunodeficiency virus; HSV-2 = herpes simplex virus type 2; PCR = polymerase chain reaction.

1. 351 women initiated the screening process. Some of the eligibility criteria were assessed by structured questioning and others by clinical assessment or laboratory testing at either the screening and/or enrollment visit. When a woman was determined to be ineligible, screening procedures were usually not completed. This is why the data in the ‘Screened’ column of Table 1 were not always collected for all 351 women.

2. Includes women who had some primary schooling but did not complete it. The same applies to secondary schooling.

3. Enrolled women reported informal trade/small business (37/71), employment by tea or coffee company (15/71), cleaning/cooking (6/71), construction (3/71), hairdressing (2/71), and other (8/71). Nine enrolled women reported to have exchanged sex for money or goods in the past year.

4. Women could report more than one method.

5. One slide was unreadable for the randomised population.

### Adherence with ring use and spontaneous ring expulsions

A single ring expulsion in the previous ring use period was reported at 51 of the 416 ring removal visits, and 2–4 expulsions in the previous ring use period at an additional seven visits. Of the 58 ring use periods during which at least one expulsion took place, the most recent expelled ring was reported to have come out ‘on its own’ 45 times (most commonly during or after sex (15), during urination (12), or during defecation (11)); to have been removed by the woman herself 13 times (because it was causing discomfort (4), it was perceived to be incorrectly placed (3), the partner wanted it taken out (1), or for other reasons (5)); or to have been removed by a husband twice (reasons unknown). In focus group discussions, women indicated that they could generally feel the ring coming out and intercept it (data not shown). Women reinserted the ring at home after 37 of the 58 most recent expulsions (in 31 cases within three hours and in six cases within 3–12 hours), or attended the study clinic to have the ring reinserted or replaced after 21 expulsions (within 3–12 hours in eight cases, within 12–24 hours in five cases, in 2–5 days in five cases, and unknown in three cases). Most women who reinserted the ring at home rinsed it with plain water prior to reinsertion (reported for 32 of the 37 expulsions after which reinsertion at home took place), and none of them used soap.

### Pregnancy incidence

There were no incident pregnancies during the trial.

### Safety

There were no incident serious adverse events, serious social harms, or early discontinuations of ring use for safety reasons during the trial. Sixty one women agreed to be tested for HIV at the last ring removal visit and none of them tested positive.

Self-reported urogenital symptoms were uncommon at baseline and at ring removal visits (Table 2), with one exception: six continuous users (10%) complained about lower abdominal pain during at least one ring removal visit compared to no intermittent users (*P* = 0.013). Clinician-observed signs during pelvic and bimanual examinations were rare at baseline and during follow-up except for the presence of blood at the cervical os (Table 2). The percentage of women with incident urogenital symptom(s) or sign(s) during at least one ring removal visit did not statistically significantly differ between intermittent and continuous users (18% versus 28%; *P* = 0.280). All women opted for VIA at the last ring removal visit and all results were normal.

**Table 2:** Urogenital self-reported symptoms and clinician-observed signs (not associated with a laboratory-confirmed infection at the same visit) by randomisation group.

Urogenital symptoms/signs recorded at baseline and ring removal visits^1^^,^ ^2^^,^ ^3^	Baseline (N = 120)	Intermittent use of three rings (N = 60)	Continuous use of four rings (N = 60)	*P*-value^4^
Total n (%) of randomised women with symptom(s) or sign(s) at the baseline visit	18 (15)	NA	NA	
Total n (%) of women with incident symptom(s) or sign(s) during at least one ring removal visit	NA	11/60 (18.3)	17/60 (28.3)	0.280
*Self-reported urinary symptoms*				
Burning when passing urine	2 (1.7)	1/59 (1.7)	4/59 (6.8)	0.364
Genital burning	1 (0.8)	3/59 (5.1)	2/60 (3.3)	0.679
Frequent urination or urgent need	0 (0.0)	1/60 (1.7)	0/60 (0.0)	1.000
*Self-reported vaginal symptoms*				
Genital itching	2 (1.7)	1/59 (1.7)	2 /59 (3.4)	1.000
Lower abdominal pain	3 (2.5)	0/59 (0.0)	6/58 (10.3)	0.013
Abnormal vaginal discharge	1 (0.8)	0/60 (0.0)	0/59 (0.0)	
Pain during sex	1 (0.8)	0/59 (0.0)	0/60 (0.0)	
*Clinician-observed signs*				
Vulvovaginal lesion/pustule	0 (0.0)	1/60 (1.7)	1/60 (1.7)	1.000
Vaginal abnormal/unusual discharge	11 (9.2)	3/56 (5.4)	1/53 (1.9)	0.619
Vaginal erythema or ulceration or laceration or abrasion or peeling or petechiae or ecchymosis or condylomata or oedema or cysts	0 (0.0)	1/60 (1.7)	0/60 (0.0)	1.000
Vaginal vesicles	0 (0.0)	0/60 (0.0)	1/60 (1.7)	1.000
Cervical abnormal/unusual discharge	1 (0.8)	2/60 (3.3)	0/59 (0.0)	0.496
Cervical erythema, ulceration, laceration, abrasion, peeling, petechiae, ecchymosis, vesicles, condylomata, oedema, cysts	1 (0.8)	0/59 (0.0)	1/60 (1.7)	1.000
Cervical os blood present	0 (0.0)	3/60 (5.0)	10/60 (16.7)	0.075
Adnexal tenderness	1 (0.8)	0/60 (0.0)	0/59 (0.0)	

NA = not applicable

1. Number of women with incident self-reported symptom or clinician-observed sign at one or more ring removal visits (%).

2. Women could report one or more symptoms, and study physicians could observe one or more signs, per ring removal visit.

3. Includes self-reported urogenital and vaginal symptoms.

4. Fisher’s exact test comparing intermittent and continuous users.

**Fig 2:**
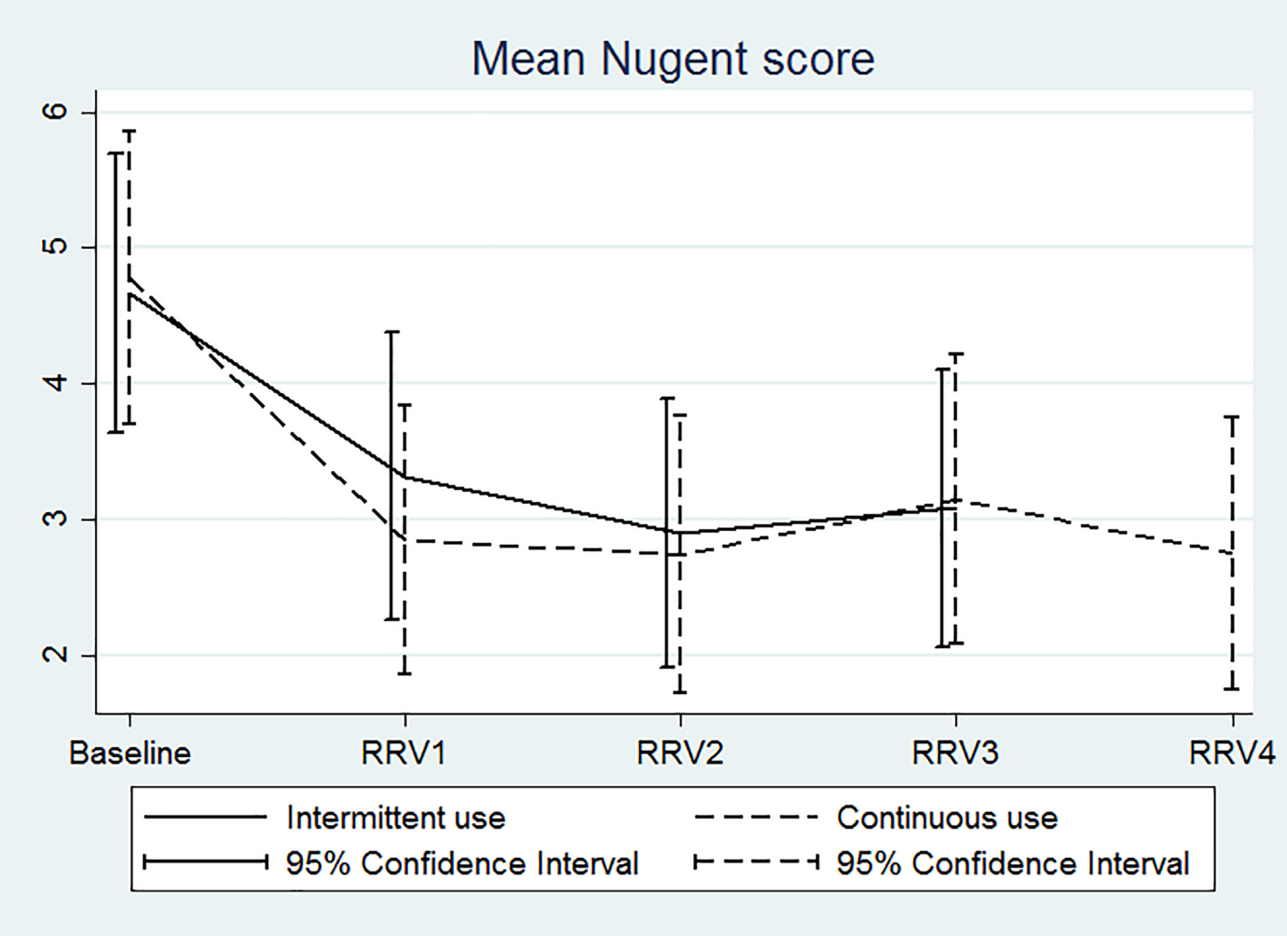
Mean Nugent score over time by randomisation group. X-axis: RRV = ring removal visit.Y-axis: Mean Nugent score for intermittent users (solid line) and continuous users (dashed line) with bars indicating 95% confidence intervals. The mean Nugent score for the 60 women in each randomisation group was calculated at baseline and at each ring removal visit. The intermittent users used three rings and therefore had three ring removal visits, whereas the continuous users had four.

The baseline prevalence of BV by Nugent score in the enrolled population was 48%. The percentages of women with incident BV at one or more ring removal visits were not statistically significantly different in the two randomisation groups (Table 3). Fig 2 shows that the mean Nugent scores of women in both groups improved with duration of ring use. In contrast, while the baseline prevalence of vaginal yeasts was only 5% in the enrolled population, the percentages of women with incident vaginal yeasts at one or more ring removal visits were high: 22% in intermittent users and 27% in continuous users (*P* = 0.666; Table 3). Symptomatic vaginal yeast cases were more common in the continuous than intermittent users (*P* = 0.031). The baseline prevalence of trichomoniasis in the enrolled population was 8%, and the percentages of women with incident infections at one or more ring removal visits were 6% in intermittent users and 7% in continuous users.

**Table 3:** Laboratory-confirmed bacterial vaginosis, vaginal yeasts and trichomoniasis by randomisation group.

Incident infection between first ring insertion and last ring removal ^1^	Intermittent use of three rings	Continuous use of four rings	*P*-value^2^
Bacterial vaginosis (Nugent 7–10)^3^	4 / 24 (16.7)	8 / 24 (33.3)	0.318
Asymptomatic	4 / 24 (16.7)	7 / 24 (29.2)	0.494
Symptomatic^4^	0 / 24 (0.0)	1 / 24 (4.2)	1.000
Vaginal yeasts (wet mount)^3^	13 / 58 (22.4)	15 / 56 (26.8)	0.666
Asymptomatic	12 / 58 (20.7)	8 /56 (14.3)	0.462
Symptomatic^4^	1 / 58 (1.7)	7 / 56 (12.5)	0.031
Trichomoniasis (wet mount)^3^	3 / 55 (5.5)	4 /56 (7.1)	1.000
Asymptomatic	2 / 55 (3.6)	3 / 56 (5.4)	1.000
Symptomatic^4^	1 / 55 (1.8)	1 / 56 (1.8)	1.000

1. Number of women with positive test result at one or more ring removal visits / Number of women who were negative for that infection at baseline (%).

2. Fisher’s exact test.

3. To incident BV also applies: a Nugent score of 7–10 after a previous Nugent score of 0–6.

4. Symptomatic: woman reported a urogenital symptom(s), or clinician observed a urogenital sign(s), at the same visit as the positive laboratory test result.

Table 4 shows AEs that were reported by women when they were asked if they had experienced any AE since the previous visit prior to any structural AE assessments. The number of women reporting one or more AEs did not significantly differ between the randomisation groups (Table 4). Among AEs that were reported at least twice during the study, menorrhagia was reported by more continuous than intermittent users (nine women versus two women; *P* = 0.053), with a rate of 1.1 and 3.8 per 100 rings, respectively (*P* = 0.120); there were no other statistically significant differences between the groups. The severity and relatedness of reported AEs were not statistically significantly different between groups either. None of the AEs were judged by a study physician to be definitely related to ring use and only four were judged to be probably related (Table 4). One social harm was reported during the entire trial. This participant had not informed her husband about her trial participation, and he was upset about her not informing him when he felt the ring during sex. The issue was resolved after couple counselling by study staff and the participant continued ring use.

**Table 4:** Spontaneously reported^1^ adverse events: comparison by group for the total ring use period and per 100 used rings.

Adverse events^2^	Total ring use period			Rate per 100 rings^3^		
**Number of women (%) reporting AEs**	**Intermittent(N = 60)**	**Continuous(N = 60)**	***P***^4^	**Intermittent**	**Continuous**	***P***^8^
At least one AE	23 (38.3)	32 (53.3)	0.142	12.8	13.3	0.876
One AE	12 (20.0)	11 (18.3)	1.000	6.7	4.6	0.369
Two AEs	8 (13.3)	10 (16.7)	0.799	4.4	4.2	0.892
Three AEs	2 (3.3)	9 (15.0)	0.053	1.1	3.8	0.120
Four AEs	1 (1.7)	1 (1.7)	1.000	0.6	0.4	0.839
Five AEs	0 (0.0)	1 (1.7)	1.000	0.0	0.4	NA
**Number of AEs (%) judged**	**(N = 38)**	**(N = 67)**	***P***^5^			
Mild	13 (34.2)	23 (34.3)	1.000			
Moderate	25 (65.8)	44 (65.7)				
**Not related**	3 (7.9)	7 (10.5)	0.308			
**Unlikely related**	16 (42.1)	19 (28.4)				
**Possibly related**	18 (47.4)	38 (56.7)				
**Probably related^**6**^**	1 (2.6)	3 (4.5)				
**Definitely related**	0 (0.0)	0 (0.0)				
**Number of women (%) reporting AEs that were reported twice or more often**^**7**^	**(N = 60)**	**(N = 60)**	***P***^4^	**Intermittent**	**Continuous**	***P***^8^
Respiratory tract infection	7 (11.7)	4 (6.7)	0.529	3.9	1.7	0.176
Headache	6 (10.0)	11 (18.3)	0.295	3.3	4.6	0.530
Back pain	3 (5.0)	8 (13.3)	0.204	1.7	3.3	0.306
Menorrhagia	2 (3.3)	9 (15.0)	0.053	1.1	3.8	0.120
Diarrhoea	1 (1.7)	6 (10.0)	0.114	0.6	2.5	0.164
Malaria	2 (3.3)	4 (6.7)	0.679	1.1	1.7	0.640
Vaginal haemorrhage	3 (5.0)	1 (1.7)	0.619	1.7	0.4	0.230
Nausea	0 (0.0)	4 (6.7)	0.119	0.0	1.7	NA
Vertigo	2 (3.3)	5 (8.3)	0.439	1.1	2.1	0.452
Abdominal pain	1 (1.7)	3 (5.0)	0.619	0.6	1.3	0.483
Asthma	1 (1.7)	1 (1.7)	1.000	0.6	0.4	0.839
Wound	1 (1.7)	1 (1.7)	1.000	0.6	0.4	0.839
Pustule	1 (1.7)	1 (1.7)	1.000	0.6	0.4	0.839

AE = adverse event. The combination of prolonged painful menses and lower abdominal pain is coded as menorrhagia. Painful menses, prolonged menses, and heavy menstrual flow are coded as menorrhagia. Lower abdominal pain is coded as abdominal pain. Vaginal bleeding and spotting are coded as vaginal haemorrhage. Amoebiasis and intestinal parasitosis are coded as diarrhoea. Cough, flu and tonsillitis are coded as respiratory tract infection. Dizziness is coded as vertigo. The combination of nausea and vomiting is coded as nausea.

1. AEs that are not captured under the structurally collected urogenital symptoms and signs, or laboratory confirmed reproductive tract infections, which were presented in Tables 2 and 3.

2. Number of women who reported the AE (%), unless indicated otherwise.

3. Numerator: number of women reporting AEs; denominator: number of women multiplied with three (180) for intermittent users and number of women multiplied with four (240) for continuous users.

4. Fisher’s exact test comparing the proportion of women in the intermittent versus continuous use group.

5. Fisher’s exact test comparing the proportions of total AEs that were mild versus moderate, or not related/unlikely related versus possibly/probably related between study groups.

6. Intermittent users: prolonged menses; Continuous users: back pain x2 and vertigo.

7. Single presence AEs in intermittent users: loss of appetite, fever, urine tract infection, and abscess leg; in continuous users: acne, breast pain, allergic rhinitis, muscle cramp and itching of the vulva.

8. Poisson regression comparing AE rates in each study group.

### Cycle control

At the final ring removal visit, more than half of the continuous users (57%) reported by structured questioning to have had no bleeding days during ring use compared to 10% of the intermittent users. Only one of the women who had not had any bleeding (a continuous user) reported that this was a problem; she was worried that she ‘might have a tumour in her uterus.’ Participants were also asked by structured questioning if they experienced any vaginal bleeding since their last visit that they did not attribute to menses or withdrawal bleeding. Such bleeding was reported by only three intermittent users and one continuous user at their first ring removal visit, one intermittent user at her second ring removal visit, and no-one thereafter.

### Acceptability

We asked women at the enrolment visit, after they had touched and seen a vaginal ring but prior to ring insertion, what concerns they had, if any, about using the ring using structured questioning. The top five worries reported were that the main partner might not like the ring (reported by 13% of women), or the ring might come out spontaneously (11%), be uncomfortable during sex (9%), cause infection (8%), or not adequately protect against pregnancy (8%). However, at the final ring removal visit, a total of only four women still reported the top two concerns (two women each). At the final ring removal visit, the majority of women reported that they had no problems inserting and removing the ring (99%), never felt the ring during daily activities (96%), and never felt the ring during sex (83%). They thought that the ring made sex feel better (88%) and that it increased vaginal lubrication (75%), which was considered a positive attribute. About half (52%) of the women said that their male partner felt the ring during sex, but that he either felt indifferent about that (33%) or liked the way it felt (19%).

## Discussion

All clinical trials and post-licensure studies to date have shown that the user effectiveness of NuvaRing® is equivalent to that of combined oral contraceptive pills [2–4, 17, 18]. Our study was not designed to confirm contraceptive effectiveness, but we did not have any incident pregnancies. While deliberate ring removals were rare, our participants reported spontaneous expulsions in 14% of the ring use periods. This is in agreement with other studies that have reported spontaneous expulsions in 4–20% of ring use periods [4]. About half of the participants who experienced an expulsion rinsed and reinserted the ring at home within three hours as instructed by study staff, but the other half either reinserted at home or in the study clinic more than three hours later. Not all of these women refrained from sex or used condoms for seven days as instructed, and they were therefore at risk of pregnancy. If vaginal rings are introduced in public clinics in Rwanda, we recommend pro-active planning for spontaneous expulsions.

Studies in Europe, North America and India have shown that there are no differences in the types and frequencies of systemic side effects in NuvaRing® users compared to oral contraceptive users [2–4, 17–21]. Some of the systemic side effects that were commonly reported in those studies, and are often attributed to oestrogens, were either not reported at all by our Rwandan participants (emotional liability, breast tenderness, and acne) or were infrequently reported (nausea). It is likely that these reflect differences in reporting rather than actual differences in prevalence, perhaps due to cultural influences. Headaches were commonly reported in all studies including ours.

The above-mentioned studies also showed that the prevalence of local side effects in NuvaRing® users is low overall but higher than in oral contraceptive users [2–4, 17–21]. The most frequently reported local side effects in these studies were vaginitis, unusual vaginal discharge, vaginal bleeding (in most cases spotting), and device-related events such as foreign body sensation. A review of the literature up to 2012 concluded that local side effects were the main reason for ring use discontinuation in those studies [4]. In our study, the prevalence of local side effects was low in both study groups despite the fact that we asked specific questions about them at each ring removal visit. The only exception was lower abdominal pain, which was reported by no intermittent and six continuous users; this was also the only local side effect that was reported significantly more often by continuous than intermittent users.

Our study was unique in that we conducted pelvic examinations and tested for STIs and vaginal infections at regular intervals, and were therefore not limited to self-reported vaginal symptoms to assess the effect of NuvaRing® on the cervicovaginal environment. We found that clinical signs during pelvic examinations, and trichomonads on wet mount, were uncommon in both groups. At baseline, the overall prevalence of BV by Nugent score in the enrolled population was high (48%) and that of yeasts on wet mount low (5%). Mean Nugent scores improved in both groups during ring use, but the percentages of women with yeasts on wet mount during at least one ring removal visit was high, with no significant difference between the two groups (22% in intermittent users and 27% in continuous users). The presence of yeasts on wet mount was usually not accompanied by participant-reported symptoms (only in 8 of a total of 28 cases), but symptomatic cases were more common in continuous (7 cases) than in intermittent users (one case). Veres et al also showed an improvement of the vaginal microbiota over three cycles of ring use [22], whereas Davies et al did not find a change in BV prevalence during continuous ring use over 56 days [23]. Furthermore, Oddsson et al observed an increase in *Candida* vaginitis during 13 weeks of ring use [19], whereas this was not observed by Veres et al [22]. An *in vitro* study demonstrated that yeast cells are able to adhere to NuvaRing®, with *Candida glabrata* showing the highest and *Candida albicans* the lowest adherence capacity [24]. A recent systematic review of the literature showed that combined oral contraceptives, and to a lesser extent progestin-only injectables, are associated with a reduced risk of BV, but that combined oral contraceptives are also associated with an increased risk of vaginal candidiasis [25]. The NuvaRing® is a combined hormonal method and our BVs and vaginal yeasts results are consistent with those of combined oral contraceptives in this systematic review. Unfortunately, providers and (potential) users are often not aware of these effects.

An important aspect of acceptability is cycle control. Unfortunately, our data did not capture cycle control reliably. First, all our participants were new ring users and it generally takes several rings for new bleeding patterns to settle [2–4]. Second, women were asked about bleeding at each ring removal visit just before their next withdrawal bleed would have started (typically 1–3 days after ring removal) [2–4] and a month after their previous withdrawal bleed; the potential for recall bias was therefore high. Past studies suggest that cycle control in NuvaRing® users is good and even superior to that of combined oral contraceptive users [2–4]. Our data do not contradict this because few women reported bleeding-related AEs. A study comparing 28, 49, 91, and 364 day cycles with each individual ring used for three weeks showed that the median number of bleeding days decreased with fewer scheduled ring-free days but that this was accompanied by an increase in spotting days [26]. More than half (57%) of the continuous users in our study reported to have had no bleeding at all during their entire ring use period compared to only 10% of the intermittent users, but we cannot draw any conclusions about spotting with our data.

In all studies to date, including ours, women reported that inserting and removing the NuvaRing® is easy [2–4]. Studies also consistently report that the majority of women do not feel the ring during daily activities or sex, and that male partners sometimes do feel the ring during sex but are generally not bothered by it [2–4]. Our finding of the ring increasing vaginal wetness, which the participants considered to be a positive attribute, was reported by only one other study [22]. We suspect that this and other sexual function and pleasure attributes are not routinely assessed. More detailed findings about acceptability, sexual function and sexual pleasure from our study will be published separately.

In conclusion, in our study, the NuvaRing® was successfully used by a population with above average risk of HIV and unintended pregnancies, which is an ideal population for roll-out of future multipurpose rings. Both intermittent and continuous NuvaRing® use were safe, improved Nugent scores over time, and were acceptable. Depo Provera® is potentially associated with an increased risk of HIV acquisition, whereas combined hormonal contraceptive pills are not [11]. It therefore seems likely that other combined hormonal contraceptive methods, including NuvaRing®, are not associated with increased HIV acquisition risk either, although this remains to be studied. For all of these reasons, as well as an ongoing unmet need for family planning in Rwanda, we recommend the addition of combined hormonal contraceptive vaginal rings to the contraceptive method mix in Rwanda. However, attention should be paid to ring expulsions and to a potential increased risk of vaginal candidiasis.

## Supporting information

S1 Fig CONSORT checklist. (PDF)

S2 Fig Approved study protocol. (PDF)

## References

[1] Brache V, Faundes A. Contraceptive vaginal rings: a review. Contraception. 2010; 82: 418–427. doi: 10.1016/j.contraception.2010.04.012 20933115 10.1016/j.contraception.2010.04.012

[2] Roumen FJ. Review of the combined contraceptive vaginal ring, NuvaRing. Ther Clin Risk Manag. 2008; 4: 441–451. 18728840 10.2147/tcrm.s1964 PMC2504064

[3] Shimoni N, Westhoff C. Review of the vaginal contraceptive ring (NuvaRing®). J Fam Plann Reprod Health Care. 2008; 34: 247–250. doi: 10.1783/147118908786000370 18854070 10.1783/147118908786000370

[4] Roumen FJME, Mishell DR. The contraceptive vaginal ring, NuvaRing ®, a decade after its introduction. Eur J Contracept Reprod Health Care. 2012; 17: 415–427. doi: 10.3109/13625187.2012.713535 23113828 10.3109/13625187.2012.713535

[5] Carr SL, Gaffield ME, Dragoman MV, Phillips S. Safety of the progesterone-releasing vaginal ring (PVR) among lactating women: A systematic review. Contraception. 2016; 94: 253–261. doi: 10.1016/j.contraception.2015.04.001 25869631 10.1016/j.contraception.2015.04.001

[6] Cleland J, Machiyama K. Unmet need for family planning: past achievements and remaining challenges. Semin Reprod Med. 2015; 33: 11–16. doi: 10.1055/s-0034-1395273 25565506 10.1055/s-0034-1395273

[7] Baeten JM, Palanee-Phillips T, Brown ER, Schwartz K, Soto-Torres LE, Govender V, et al Use of a vaginal ring containing dapivirine for HIV-1 prevention in women. N Engl J Med. 2016; 375: 2121–2132. doi: 10.1056/NEJMoa1506110 26900902 10.1056/NEJMoa1506110 PMC4993693

[8] Nel A, van Niekerk N, Kapiga S, Bekker LG, Gama C, Gill K, et al Safety and efficacy of a dapivirine vaginal ring for HIV prevention in women. N Engl J Med. 2016; 375: 2133–2143. doi: 10.1056/NEJMoa1602046 27959766 10.1056/NEJMoa1602046

[9] Fernández-Romero JA, Deal C, Herold BC, Schiller J, Patton D, Zydowsky T, et al Multipurpose prevention technologies: the future of HIV and STI protection. Trends Microbiol. 2015; 23: 429–436. doi: 10.1016/j.tim.2015.02.006 25759332 10.1016/j.tim.2015.02.006 PMC4490993

[10] Westhoff CF. The recent fertility transition in Rwanda. Population and Development Review. 2013; 38: 169–178.

[11] Polis CB, Curtis KM, Hannaford PC, Phillips SJ, Chipato T, Kiarie JN, et al An updated systematic review of epidemiological evidence on hormonal contraceptive methods and HIV acquisition in women. AIDS. 2016; 30: 2665–2683. doi: 10.1097/QAD.0000000000001228 27500670 10.1097/QAD.0000000000001228 PMC5106090

[12] Nel AM, Coplan P, van de Wijgert JH, Kapiga SH, von Mollendorf C, Geubbels E, et al Safety, tolerability, and systemic absorption of dapivirine vaginal microbicide gel in healthy, HIV-negative women. AIDS. 2009; 23: 1531–1538. doi: 10.1097/QAD.0b013e32832c413d 19550287 10.1097/QAD.0b013e32832c413d

[13] Schurmans C, De Baetselier I, Kestelyn E, Jespers V, Delvaux T, Agaba SK, et al The ring plus project: safety and acceptability of vaginal rings that protect women from unintended pregnancy. BMC Public Health. 2015; 15: 348 doi: 10.1186/s12889-015-1680-y 25880636 10.1186/s12889-015-1680-y PMC4404010

[14] Nugent RP, Krohn MA, Hillier SL. Reliability of diagnosing bacterial vaginosis is improved by a standardized method of Gram stain interpretation. J Clin Microbiol. 1991; 29: 297–301. 1706728 10.1128/jcm.29.2.297-301.1991 PMC269757

[15] Brown EG, Wood L, Wood S. The medical dictionary for regulatory activities (MedDRA). Drug-Safety. 1999; 20: 109 10082069 10.2165/00002018-199920020-00002

[16] National Institute of Statistics of Rwanda (NISR), Ministry of Health (MOH) Rwanda, and ICF International. Rwanda Demographic and Health Survey 2010. Calverton, MD, USA, 2012: NISR/Rwanda, MOH/Rwanda, and ICF International Accessed on 9 February 2016 at: https://dhsprogram.com/pubs/pdf/FR316/FR316.pdf.

[17] Roumen FJ, Apter D, Mulders TM, Dieben TO. Efficacy, tolerability and acceptability of a novel contraceptive vaginal ring releasing etonogestrel and ethinyl oestradiol. Hum Reprod. 2001; 16: 469–475. 11228213 10.1093/humrep/16.3.469

[18] Dieben TO, Roumen FJ, Apter D. Efficacy, cycle control, and user acceptability of a novel combined contraceptive vaginal ring. Obstet Gynecol. 2002; 100: 585–593. 12220783 10.1016/s0029-7844(02)02124-5

[19] Oddsson K, Leifels-Fischer B, de Melo NR, Wiel-Masson D, Benedetto C, Verhoeven CH, Dieben TO. Efficacy and safety of a contraceptive vaginal ring (NuvaRing) compared with a combined oral contraceptive: a 1-year randomized trial. Contraception. 2005; 71: 176–182. doi: 10.1016/j.contraception.2004.09.001 15722066 10.1016/j.contraception.2004.09.001

[20] Sabatini R, Cagiano R. Comparison profiles of cycle control, side effects and sexual satisfaction of three hormonal contraceptives. Contraception. 2006; 74: 220–223. doi: 10.1016/j.contraception.2006.03.022 16904415 10.1016/j.contraception.2006.03.022

[21] Pandit SN, Chauhan AR, Anagani M, Reddy S, Birla A, Ray SK. Multicenter Study of contraceptive vaginal ring (NuvaRing®) in normal daily practice in Indian women. J Obstet Gynaecol India. 2014; 64: 409–416. doi: 10.1007/s13224-014-0559-7 25489144 10.1007/s13224-014-0559-7 PMC4257921

[22] Veres S, Miller L, Burington B. A comparison between the vaginal ring and oral contraceptives. Obstet Gynecol. 2004; 104: 555–563. doi: 10.1097/01.AOG.0000136082.59644.13 15339769 10.1097/01.AOG.0000136082.59644.13

[23] Davies GC, Feng LX, Newton JR, Dieben TO, Coelingh-Bennink HJ. The effects of a combined contraceptive vaginal ring releasing ethinyloestradiol and 3-ketodesogestrel on vaginal flora. Contraception. 1992; 45: 511–518. 1623721 10.1016/0010-7824(92)90163-n

[24] Camacho DP1, Consolaro ME, Patussi EV, Donatti L, Gasparetto A, Svidzinski TI. Vaginal yeast adherence to the combined contraceptive vaginal ring (CCVR). Contraception. 2007; 76: 439–443. doi: 10.1016/j.contraception.2007.07.012 18061701 10.1016/j.contraception.2007.07.012

[25] van de Wijgert JH, Verwijs MC, Norris Turner A, Morrison CS. Hormonal contraception decreases bacterial vaginosis but oral contraception may increase candidiasis: implications for HIV transmission. AIDS. 2013; 27: 2141–2153. doi: 10.1097/QAD.0b013e32836290b6 23660575 10.1097/QAD.0b013e32836290b6

[26] Miller L, Verhoeven CH, Hout J. Extended regimens of the contraceptive vaginal ring: a randomized trial. Obstet Gynecol. 2005; 106: 473–482. doi: 10.1097/01.AOG.0000175144.08035.74 16135576 10.1097/01.AOG.0000175144.08035.74

